# Gcg-XTEN: An Improved Glucagon Capable of Preventing Hypoglycemia without Increasing Baseline Blood Glucose

**DOI:** 10.1371/journal.pone.0010175

**Published:** 2010-04-14

**Authors:** Nathan C. Geething, Wayne To, Benjamin J. Spink, Michael D. Scholle, Chia-wei Wang, Yong Yin, Yi Yao, Volker Schellenberger, Jeffrey L. Cleland, Willem P. C. Stemmer, Joshua Silverman

**Affiliations:** 1 Amunix, Inc., Mountain View, California, United States of America; 2 Versartis, Inc., Mountain View, California, United States of America; Pennington Biomedical Research Center/LSU, United States of America

## Abstract

**Objective:**

While the majority of current diabetes treatments focus on reducing blood glucose levels, hypoglycemia represents a significant risk associated with insulin treatment. Glucagon plays a major regulatory role in controlling hypoglycemia *in vivo*, but its short half-life and hyperglycemic effects prevent its therapeutic use for non-acute applications. The goal of this study was to identify a modified form of glucagon suitable for prophylactic treatment of hypoglycemia without increasing baseline blood glucose levels.

**Methodology/Principal Findings:**

Through application of the XTEN technology, we report the construction of a glucagon fusion protein with an extended exposure profile (Gcg-XTEN). The *in vivo* half-life of the construct was tuned to support nightly dosing through design and testing in cynomolgus monkeys. Efficacy of the construct was assessed in beagle dogs using an insulin challenge to induce hypoglycemia. Dose ranging of Gcg-XTEN in fasted beagle dogs demonstrated that the compound was biologically active with a pharmacodynamic profile consistent with the designed half-life. Prophylactic administration of 0.6 nmol/kg Gcg-XTEN to dogs conferred resistance to a hypoglycemic challenge at 6 hours post-dose without affecting baseline blood glucose levels. Consistent with the designed pharmacokinetic profile, hypoglycemia resistance was not observed at 12 hours post-dose. Importantly, the solubility and stability of the glucagon peptide were also significantly improved by fusion to XTEN.

**Conclusions/Significance:**

The data show that Gcg-XTEN is effective in preventing hypoglycemia without the associated hyperglycemia expected for unmodified glucagon. While the plasma clearance of this Gcg-XTEN has been optimized for overnight dosing, specifically for the treatment of nocturnal hypoglycemia, constructs with significantly longer exposure profiles are feasible. Such constructs may have multiple applications such as allowing for more aggressive insulin treatment regimens, treating hypoglycemia due to insulin-secreting tumors, providing synergistic efficacy in combination therapies with long-acting GLP1 analogs, and as an appetite suppressant for treatment of obesity. The improved physical properties of the Gcg-XTEN molecule may also allow for novel delivery systems not currently possible with native glucagon.

## Introduction

Current diabetes treatment paradigms encourage aggressive insulin regimens to manage blood glucose levels within normal ranges and prevent increases in HbA1c [1]. Several studies have shown that patient outcomes would be further improved by even more aggressive insulin dosing, but hypoglycemic episodes represent a limiting adverse effect [2], [3], [4], [5]. While current treatment methods focus almost exclusively on reducing blood glucose levels, normal glucose homeostasis requires the action of multiple hormones with complementary activities in order to maintain blood glucose in a relatively constant range [6], [7]. Therefore, it is likely that treatment with multiple regulatory hormones may provide a better overall patient outcome by stabilizing blood glucose within a normal range. Such an approach may help to reduce the incidence of adverse effects such as hypoglycemia.

In particular, nocturnal hypoglycemia represents a significant unmet medical need. Whereas hypoglycemic events during the day can be recognized and treated with relative ease, nocturnal events are more problematic. At least one study has shown that the normal waking response to hypoglycemia is impaired in diabetic patients [8]. Further, up to 22% of all autopsied deaths among Australian type I diabetics under 40 years old over a 12 year period were attributed to complications of nocturnal hypoglycemia [9]. The therapeutic need is most acute among juvenile type I diabetes patients where incidence of nocturnal hypoglycemic episodes is especially high [10], [11], [12], [13].

The only currently approved drug for treatment of hypoglycemia is glucagon. Glucagon is a key hormone in glucose homeostasis and normally limits the severity of hypoglycemic events [14], [15]. However, due to its extremely short half-life (<10 minutes), the currently marketed form of glucagon is only indicated for acute hypoglycemic episodes [16]. Due to the difficulties associated with detecting nocturnal hypoglycemia, a long-lasting prophylactic form of glucagon would be preferred. Recent studies have demonstrated success in preventing nocturnal hypoglycemia using continuous infusion and slow-release depot formulations of very low dose glucagon [17]. However, these approaches are limited due to the physical properties of the glucagon peptide itself. Glucagon is highly insoluble and unstable in liquid formulations [16], limiting its application in pumps or other continuous administration strategies.

XTEN technology may represent an opportunity to optimize the glucagon peptide for prophylactic applications. The XTEN sequence has been demonstrated to controllably increase the serum half-life of peptides and proteins [18]. In addition, fusion of the XTEN sequence can increase the solubility and stability of the attached peptide, while simultaneously enabling recombinant production and improved manufacturing. The XTEN sequence has been shown to be safe and poorly immunogenic in a number of animal species. We therefore constructed a series of glucagon-XTEN fusion constructs (Gcg-XTEN) to assess the *in vivo* effects of extended glucagon exposure.

We chose to focus initially on identifying a Gcg-XTEN capable of preventing nocturnal hypoglycemia. For this indication, a specific pharmacodynamic profile is required. An ideal molecule should maintain efficacious serum levels for approximately 8–10 hours after administration to cover the patient's sleeping period. However, to reduce possible interactions with normal insulin usage in the patient during the day, the ideal construct would fall below efficacious serum levels between 10 and 12 hours after administration. An additional concern regarding long-term glucagon administration is the potential for an overall increase in serum glucose levels, which could lead to a worsening of the patient's disease state. Thus, a critical feature of the final molecule is a flat exposure profile that maintains an efficacious dose while minimizing effects on baseline glucose levels. Demonstration of efficacy without significant increase in blood glucose may also enable development of XTEN fusions capable of preventing hypoglycemia at weekly to monthly dosing intervals. Long-term resistance to hypoglycemia would allow for more aggressive insulin regimens for patients, providing improved patient outcomes while reducing the incidence of adverse effects.

## Results

### XTEN Optimization

**Figure 1:**
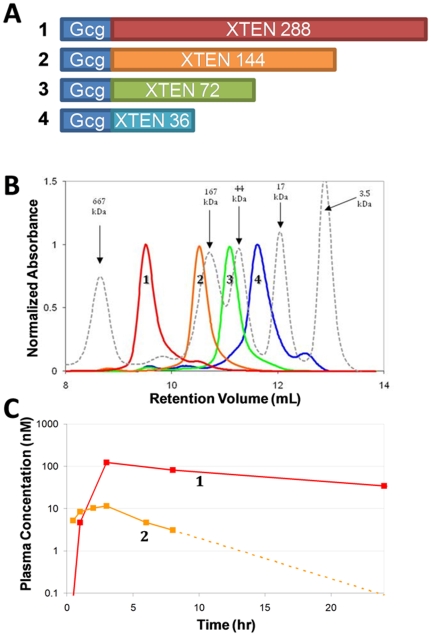
Optimizing the pharmacokinetic profile of Gcg-XTEN. (A) Schematic representations of four glucagon constructs. The length of attached XTEN in number of amino acids is indicated on each construct. (B) Size exclusion chromatography of the four purified constructs. Chromatograms are labeled and colored for each construct as in panel A. The dashed chromatogram shows the profile of a mixed reference size standard (BioRad Laboratories). The molecular weight of each reference peak is noted. A large increase in apparent molecular weight due to an increased hydrodynamic radius is typically observed for XTEN fusion proteins in this assay. (C) Pharmacokinetic profile of constructs 1–2 over 24 hours in cynomolgus monkeys. Curves are labeled and colored as in panel A. For construct 2, serum concentration at 24 hours was below limit of detection (approximately 0.1 nM), hence the dashed line approximates the slowest terminal half-life consistent with this observation. Construct 3 was also tested in parallel, but was below limit of detection at all time points, suggesting that it has a very short plasma half-life. Based on the rapid clearance of construct 3, construct 4 was not tested in animals.

In order to determine the ideal Gcg-XTEN for overnight administration, four length variants were constructed. These constructs comprised the mammalian glucagon peptide sequence with either 288, 144, 72, or 36 amino acids of XTEN sequence genetically fused to the C-terminus (Fig 1A). The four fusions covered a broad range of hydrodynamic radii, dependent on the length of the XTEN sequence attached, consistent with the expected bulking effects of the XTEN sequence (Fig 1B).

The constructs were injected subcutaneously into cynomolgus monkeys in order to determine their pharmacokinetic profiles (Fig 1C). The observed terminal half lives correlated with the length of the attached XTEN sequence. Construct 1, comprising 288 amino acids of XTEN sequence, demonstrated the longest exposure profile with a terminal half-life of approximately 9 hrs. For treatment of nocturnal hypoglycemia, however, the target exposure profile is a constant serum concentration over the first 8–10 hours followed by rapid clearance, as described above. Construct 2, comprising 144 amino acids of XTEN sequence, best approximates the desired profile and hence this XTEN length was chosen for further study.

**Figure 2:**
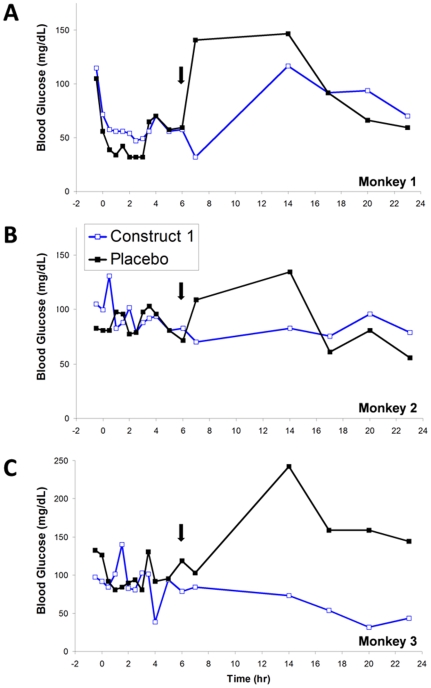
Construct 1 inhibits increase in blood glucose after end of fasting in cynomolgus monkeys. The effect of long-lived construct 1 on appetite suppression was tested in normal cynomolgus monkeys. Panels A–C show overlaid plots of blood glucose profiles after placebo or construct 1 administration for each individual animal. Solid arrows mark the time when food was returned to the animals (t = 6 hours).

While blood glucose measurements were taken at multiple timepoints after administration in the PK studies, high interanimal variability and intraanimal fluctuations prevented the observation of PD effects that could be directly correlated with the observed PK. However, potential pharmacodynamic effects of construct 1 were observed in fasted cynomolgus monkeys (Fig 2). Fasted animals were administered either 7 nmol/kg construct 1 or placebo. Food was made available *ad libitum* at six hours after dosing. Placebo treated animals showed an immediate increase in serum glucose levels, consistent with an eating response. In contrast, when the same animals were treated with construct 1, no increase in serum glucose after return of food was noted. This effect is consistent with suppression of appetite, which is a known biological effect of glucagon [19]. These observations suggest that construct 1 is biologically active in cynomolgus monkeys with effects that last for at least 6 hours, significantly longer than would be expected for unmodified glucagon.

**Figure 3:**
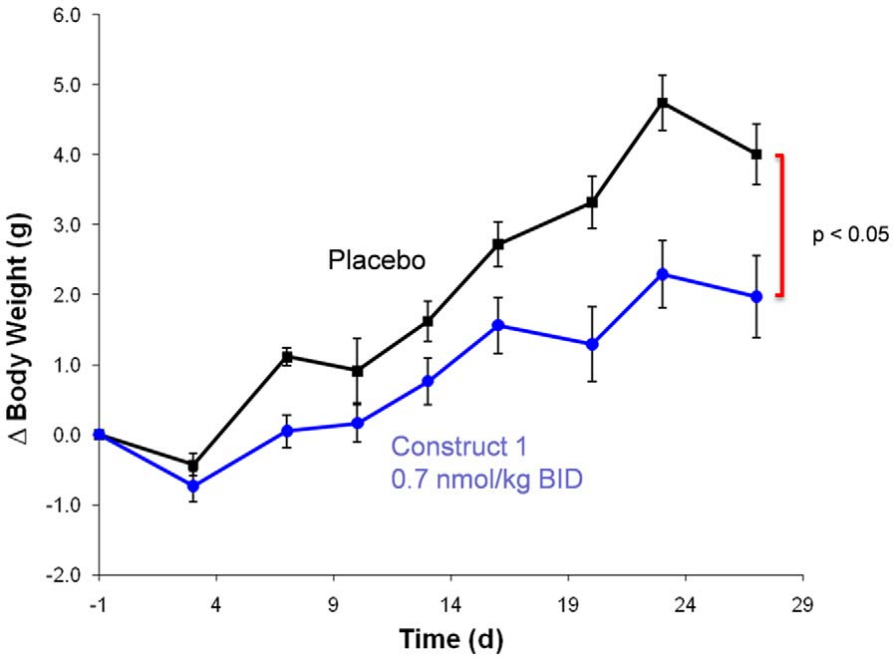
Chronic dosing of Construct 1 in Diet-Induced Obese Mice: Weight Loss. Change in body weight in Diet-Induced Obese mice over the course of 28 days continuous drug administration. Values shown are the average +/− SEM of 10 animals per group. Groups were found to be significantly different (p<0.05) by repeated measures ANOVA.

**Figure 4:**
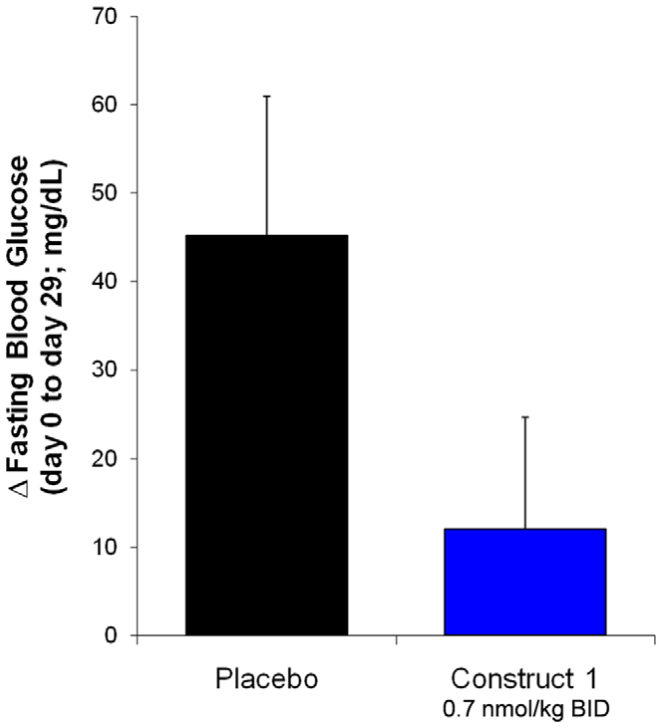
Chronic dosing of Construct 1 in Diet-Induced Obese Mice: Fasting Blood Glucose. Change in fasting blood glucose in Diet-Induced Obese mice after 28 days continuous drug administration. Values shown are the average +/− SEM of 10 animals per group. Groups were not found to be significantly different by t test.

To address concerns regarding potential elevations of blood glucose levels after chronic administration of long-lived glucagon constructs, construct 1 was administered to diet-induced obese mice for 28 days. Due to the more rapid clearance of XTEN proteins in small rodents than in larger species [18], the protein was administered twice per day to ensure sufficient exposure of the animals over the course of the study. Consistent with the potential appetite suppression effects observed in the monkeys above, continuous administration of construct 1 resulted in a significant reduction in weight gain relative to placebo treated animals (Fig 3). This effect has potential applications for treatment of a number of disease indications. Importantly, no significant change in fasting blood glucose was observed after chronic dosing of construct 1 (Fig 4), suggesting that pharmacologically active doses that do not increase baseline blood glucose may be possible in larger animals as well.

### Construction and Biophysical Characterization of Gcg-XTEN

**Figure 5:**
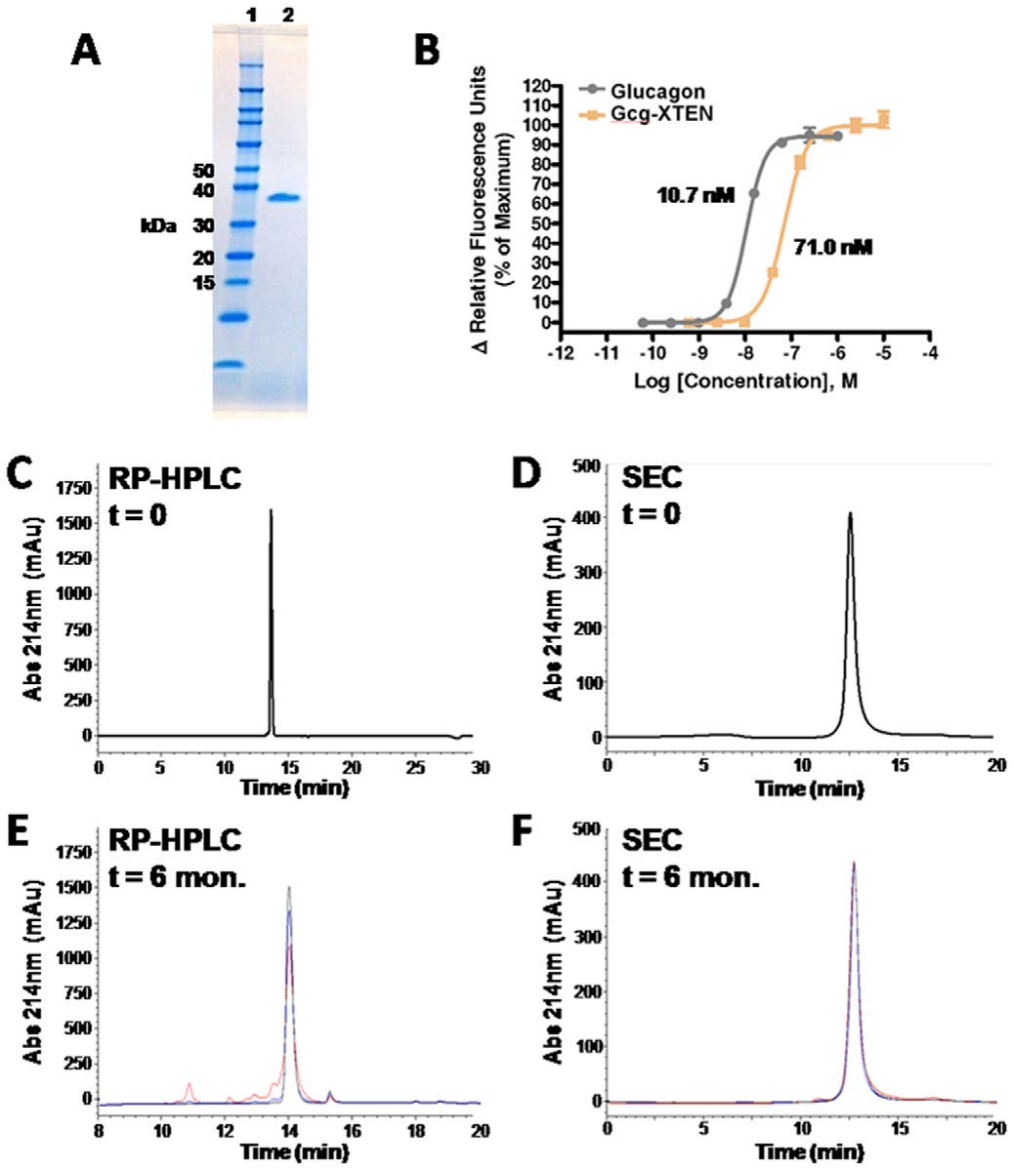
Biophysical Characterization and Stability of Gcg-XTEN. Gcg-XTEN was produced recombinantly in *E. coli* and purified to homogeneity using three column steps (see methods). (A) SDS-PAGE analysis of the purified protein product (lane 2). Molecular weight markers are shown in lane 1 with relevant size markers labeled at the left. Note that the true molecular weight of the molecule is 16305 daltons (confirmed by mass spectrometry; not shown). Slow migration in SDS-PAGE relative to globular protein standards is typical of XTEN fusion proteins due to differences in primary amino acid composition. (B) Glucagon receptor (GcgR) Ca^2+^-flux assay comparing the efficacy of Gcg-XTEN to unmodified glucagon. Calculated EC50 values for each curve fit are shown. (C) Reverse phase C18 HPLC analysis and (D) Size exclusion chromatography HPLC analysis of the purified Gcg-XTEN construct at the time of production. (E) Reverse phase C18 HPLC analysis and (F) Size exclusion chromatography HPLC analysis of Gcg-XTEN after 6 months storage at either −80°C (black), 2–8°C (blue), or 25°C (red). Note the scale is expanded in panel E to better illustrate the appearance of minor peaks at 25°C.

The XTEN sequence in construct 2 was replaced with a different sequence of identical length in order to reduce the overall charge of the molecule, thereby improving its purification properties with regard to separation from DNA and other process-related impurities (data not shown). Based on data for multiple XTEN sequences and payloads [18], no difference in the PK profile of construct 2 is expected due to the change in the XTEN sequence composition. This molecule is referred to as Gcg-XTEN. Gcg-XTEN was expressed in *Escherichia coli* and purified to homogeneity (Fig 5; see Methods). The purified protein was found to have 15% of the potency (on a molar basis) of unmodified glucagon in *in vitro* cell-based assays (Fig 5B). This activity level was found to be constant over constructs 1–4 as well (data not shown), suggesting that the apparent reduction in potency may result from steric hindrance at the C-terminus of the glucagon peptide.

In order to test the solubility and liquid stability of Gcg-XTEN, the protein was concentrated to 3.6 mM (58 mg/mL) in Tris-buffered saline at neutral pH. It is important to note that no visible aggregates or significant viscosity was noted at this stage; therefore this value should be considered a lower limit of potential solubility for Gcg-XTEN. In comparison, the solubility limit of unmodified glucagon in the same buffer was measured at 0.06 mM (0.2 mg/mL), demonstrating that the XTEN sequence confers an increase in solubility of at least 60-fold. Characterization of the Gcg-XTEN solution by reverse-phase HPLC and size exclusion chromatography indicates that the protein is homogeneous and non-aggregated in solution (Fig 5C,D). Further, the protein was found to be stable in liquid formulation for at least 6 months under refrigerated conditions (Fig 5D,E) and for approximately one month at 37°C (data not shown). Formulation optimization and excipient screening using standard methods have not yet been attempted, and may allow for even longer stability in liquid formulations.

### *In vivo* Characterization

**Figure 6:**
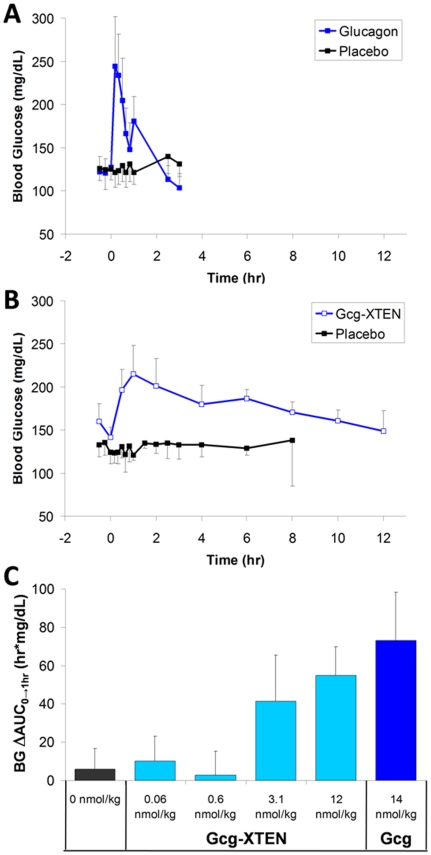
Gcg-XTEN shows extended pharmacodynamics in dogs. Glucagon (A) or Gcg-XTEN (B) was injected at 14 or 12 nmol/kg, respectively, into fasted beagle dogs (n = 4 per group) and blood glucose levels were monitored in comparison to placebo injection. (C) The difference in blood glucose area under the curve for the first hour after injection of placebo, Gcg-XTEN, or Glucagon (Gcg) relative to pre-injection baseline is shown (n = 4–8 animals per group). The dose level for each group is indicated. Values for all panels are the average values plus or minus the standard deviation.

The pharmacodynamics of Gcg-XTEN were evaluated in fasted dogs. In order to demonstrate the functionality and extended pharmacokinetic profile of the molecule, a dose of Gcg-XTEN was administered at relatively high dose (12 nmol/kg) where an increase in blood glucose levels was expected. Gcg-XTEN administration was found to rapidly increase blood glucose levels in the dog model (Fig 6). While administration of unmodified glucagon resulted in a return to baseline blood glucose levels within 2 hours (Fig 6A), animals treated with Gcg-XTEN showed elevated blood glucose levels for 10–12 hours following subcutaneous injection (Fig 6B). The increase in blood glucose levels due to Gcg-XTEN was found to be dose dependent, with doses 0.6 nmol/kg or lower showing no significant increase in blood glucose (Fig 6C). This is consistent with the results of the study of construct 1 in diet-induced obese mice, where 28 days continuous dosing of 0.7 nmol/kg yielded a slight decrease in fasting blood glucose levels relative to placebo treatment (Fig 4). To minimize potential elevation of baseline blood glucose levels, the 0.6 nmol/kg dose was selected for further study.

**Figure 7:**
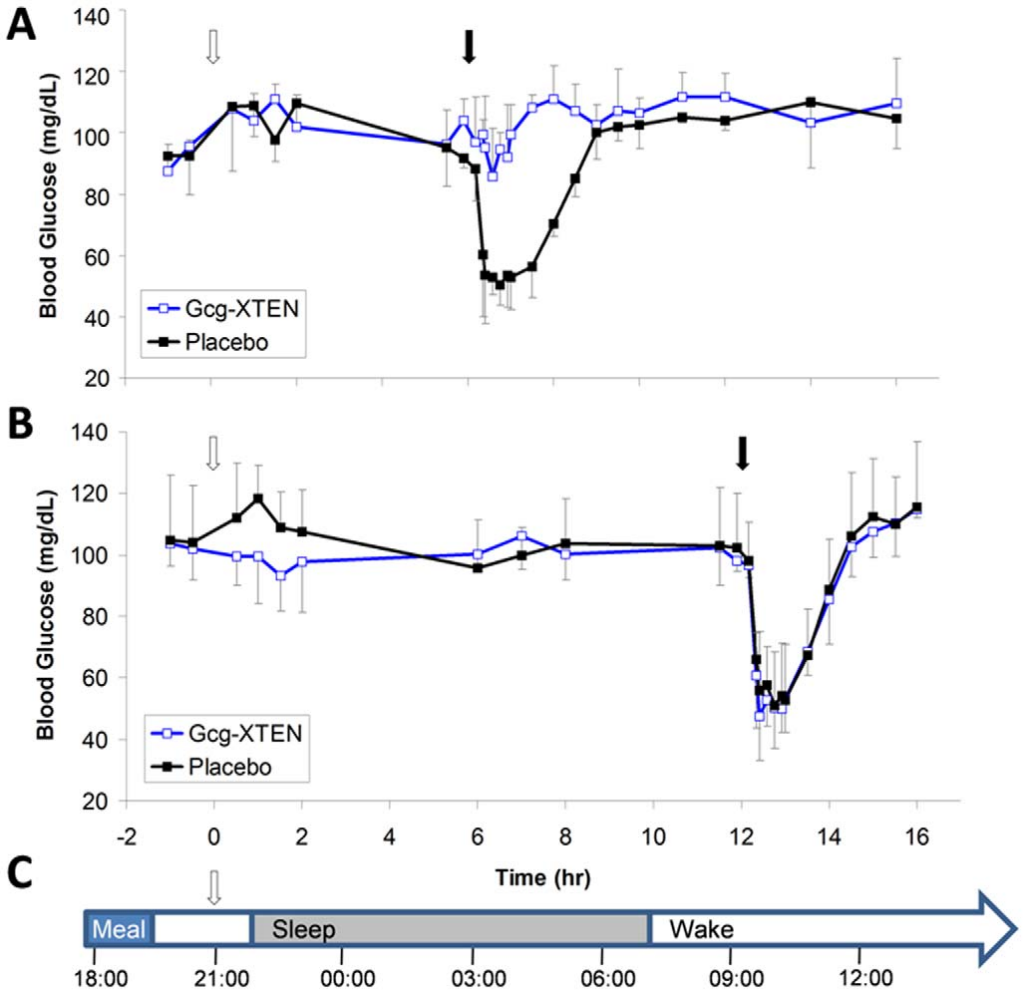
Gcg-XTEN confers temporally-controlled resistance to insulin-induced hypoglycemia in dogs. Beagle dogs were fed three hours prior to the start of the experiment and fasted thereafter. At time = 0, animals received either a dose of 0.6 nmol/kg Gcg-XTEN or placebo (open arrows). Animals (n = 4 per group) received a challenge of 0.05 U/kg insulin to induce hypoglycemia at either 6 hr (A) or 12 hr (B) after initial dose, indicated by solid arrows. Values shown are the average blood glucose plus or minus the standard deviation. (C) A hypothetical timeline for human administration. Assuming Gcg-XTEN dosing at 21:00, 6 hr post dose corresponds to 03:00 (during sleep) where protection of hypoglycemia is desired, and 12 hr post dose corresponds to 09:00 where the pharmacodynamic effect should have expired to allow for a morning meal.

To verify that Gcg-XTEN is efficacious in preventing hypoglycemia, the molecule was evaluated in an insulin-induced hypoglycemia model in dogs (Fig 7). To mimic hypothetical human usage (dosing after dinner, immediately prior to sleep), animals were fed 3 hours prior to dosing, then fasted for the remainder of the study. Animals were dosed subcutaneously with either placebo or 0.6 nmol/kg Gcg-XTEN. To demonstrate efficacy during the expected ‘sleep’ cycle, insulin was administered to induce hypoglycemia 6 hours after dosing (Fig 7A). In a separate experiment, insulin was administered to the same animals 12 hours after dosing to demonstrate that Gcg-XTEN efficacy does not persist into the expected ‘wake’ cycle (Fig 7B). Gcg-XTEN-treated animals showed a strong resistance to the hypoglycemic challenge at 6 hours after dosing, but no resistance to a challenge 12 hours after dosing. Importantly, no significant difference in the blood glucose of Gcg-XTEN treated animals relative to placebo treatment was observed at any point except during the hypoglycemic challenge at 6 hours. While the fact that this experiment was conducted in normal dogs allows for the possibility that increased insulin secretion due to glucose counter-regulation may have suppressed baseline blood glucose in Gcg-XTEN treated animals, no significant differences in insulin levels were noted during the study (Table 1). Further, the results of chronic dosing in diet-induced obese mice reported above are consistent with this observation. Thus Gcg-XTEN may prove useful for the prevention of nocturnal hypoglycemia without elevating baseline blood glucose.

**Table 1:** Insulin Levels in Gcg-XTEN or placebo treated dogs.

	Serum Insulin Concentration^a^	
Hours post dose	7	13
Gcg-XTEN	5±4	9±2
Placebo	10±3	11±4

a Concentration values are reported as µIU/mL ± standard deviation.

## Discussion

The data presented above show that Gcg-XTEN is effective at preventing hypoglycemia according to the specifications outlined for nocturnal hypoglycemia. In particular, the pharmacodynamic profile has been tuned such that an insulin challenge at 6 hours after dosing is resisted whereas a challenge at 12 hours is unaffected. This timing can be mapped onto a hypothetical timeline of a patient's usage where Gcg-XTEN was administered immediately prior to sleep at 21:00. The insulin challenge at 6 hours post-dose would therefore correspond to 03:00 (Fig 7C) which represents a natural nadir in blood glucose levels [20]. The insulin challenge at 12 hours post-dose would similarly correspond to 09:00 on the same hypothetical timeline. At this time, most patients would have awakened, eaten a meal, and had their first insulin injection. The observed lack of effect of Gcg-XTEN on insulin challenge at this time is therefore significant, suggesting that no residual activity remains that might interfere with the patient's normal daily insulin regimen.

It is important to note that the dogs used in this study had normal glycemic control and hence may be able to achieve euglycemia in the presence of low levels of glucagon activity via insulin counter-regulation. Although no significant differences in insulin levels were noted in the study, it is possible that the residual activity threshold for blood glucose elevation may be significantly higher in this model than in diabetic animals. In addition, sustained efficacy over multiple doses has not yet been demonstrated. Further, long-term studies in glycemic clamped animal models and preclinical models of diabetes are warranted to better define the potential therapeutic window between hypoglycemia resistance and hyperglycemia. These data will be important in designing clinical studies in type 1 diabetes patients to provide the desired duration of pharmacodynamic effect without increases in blood glucose.

While this report has focused on modifying glucagon to achieve hypoglycemia resistance, a large number of other hormones and signaling peptides have short half-lives and may also be amenable to optimization by XTEN addition. The recombinant nature of XTEN fusion proteins allows for rapid screening of variant constructs, allowing optimization of the pharmacokinetic and pharmacodynamic profiles for each payload molecule specific to its desired clinical indication. Thus, we expect that XTEN may enable new applications for a wide range of novel metabolic products.

The XTEN sequence confers several additional improvements to its payload peptide beyond extended pharmacokinetics. Addition of the XTEN sequence allows the fusion protein to be expressed recombinantly in *E. coli* at high yield. This is expected to result in significant reduction in the manufacturing costs of XTEN fusions relative to synthetic peptides. Further, the addition of XTEN can improve the solubility of its payload, in the case of glucagon by at least 60-fold. Our data also show that Gcg-XTEN is stable in liquid formulation for extended periods, with no sign of aggregation typically observed for hydrophobic peptides. These observations suggest that Gcg-XTEN constructs will be amenable to real-time monitoring and pump approaches. Such methods are difficult to implement with the unmodified peptide, due to the need for a lyophilized formulation. Although *in vitro* cell-based data imply that XTEN can reduce biological activity, the Gcg-XTEN *in vivo* data show comparable or better glucose mobilization on a molar dose basis relative to unmodified glucagon (Fig 3C). This discrepancy is likely due to the increased exposure of Gcg-XTEN *in vivo*, suggesting that reduced specific activity is not likely to strongly affect overall efficacy of a fusion construct. For payloads where peak dose toxicity is a concern, lower specific activity of the XTEN fusion may help to reduce the occurrence of adverse drug effects.

In addition to the above effects, a significant feature of the Gcg-XTEN molecule is the achievement of efficacy without an associated increase in blood glucose levels. While this result appears initially paradoxical, glucagon is a key signaling hormone component in the glucose counterregulatory system [14]. Similar counter-intuitive synergistic effects have been observed with a molecule capable of agonizing the glucagon and GLP-1 receptors simultaneously [21]. Sustained Gcg-XTEN levels may therefore impact a variety of signaling pathways that modulate insulin sensitivity (among other factors) leading to the observed effects. The ability to modulate the pharmacokinetic and pharmacodynamic profiles of glucagon through addition of various XTEN constructs provides a unique opportunity to characterize this poorly studied aspect of normal glucose metabolism and regulation.

While further work will be required to elucidate the precise mechanism at play, the observed efficacy without increasing baseline blood glucose levels may be highly significant in enabling novel therapies. By increasing the size of the attached XTEN sequence, achievement of up to monthly dosing intervals in humans is expected [18]. Therefore, it may be possible to create an XTEN fusion capable of conferring long-term resistance to hypoglycemia in diabetic patients without increasing long-term blood glucose levels. Such a construct could also be combined with long-acting exenatide constructs such as exenatide-LAR [22] or VRS-859 [18] to better characterize the recently reported synergy between these two payload activities. Finally, a long-acting molecule capable of preventing low blood glucose levels (and associated hunger) without significant adverse effects may represent an ideal treatment for obesity in otherwise healthy individuals.

## Materials and Methods

### Gcg-XTEN Construction

Gcg-XTEN gene construction was performed essentially as described in ref [18]. The final construct comprised the gene encoding the cellulosome anchoring protein cohesion region cellulose binding domain (CBD) from *Clostridium thermocellum* (accession #ABN54273), a tobacco etch virus (TEV) protease recognition site (ENLYFQ), the glucagon sequence, and the appropriate XTEN sequence under control of a T7 promoter. The protein was expressed and purified essentially as described for E-XTEN in ref [18]. Briefly, protein expression was induced by addition of 1 mM IPTG to a log phase culture of BL21-Gold (DE3) *E. coli* carrying the expression plasmid. TEV protease was added to heat-treated cell lysate containing Gcg-XTEN to remove the CBD sequence and generate the native N-terminus of glucagon. The cleaved protein was then purified over DE52, MacroCap Q, and Butyl Sepharose FF columns. The final material was formulated in 20 mM Tris pH 7.5, 135 mM NaCl and sterile filtered using a 0.22 micron filter. Expression was determined to be approximately 7 mg protein per gram wet cell weight (∼100 mg/L at final OD ∼4) and overall purification yield was approximately 60%.

### Animal Welfare Statement

Animal welfare for the studies described herein were performed in compliance with the U.S. Department of Agriculture's (USDA) Animal Welfare Act (9 CFR Parts 1, 2 and 3). The Guide for the Care and Use of Laboratory Animals, Institute of Laboratory Animal Resources, National Academy Press, Washington, D.C., 1996, was followed in all cases. Testing facilities maintain an Animal Welfare Assurance statement with the National Institutes of Health, Office of Laboratory Animal Welfare. In order to ensure compliance, protocols were approved by the Institutional Animal Care and Use Committee (IACUC) before the initiation of treatment. No procedures or test articles were used which would cause more than momentary pain or distress to the animals. Detailed protocols, in-life summaries, and study reports are on file at Amunix, Inc.

### Blood glucose effects after fasting in cynomolgus monkeys

The effect of long-lived construct 1 on appetite suppression was tested in normal cynomolgus monkeys. Animals were fasted for 12 hours prior to dosing and 6 hours following dosing. At time 0, animals received a randomized dose of either 7 nmol/kg of construct 1 or placebo. Blood glucose was measured by hand-held glucometer at designated times throughout the study. In a second phase, the same animals were treated with the alternate test article according to the same protocol.

### Chronic Dosing in Diet-Induced Obese Mice

The effects of chronic dosing of construct 1 were tested in male C57BL/6J Diet-Induced Obese (DIO) Mice, age 10 weeks old. Mice raised on a 60% high fat diet were randomized into treatment groups (n = 10 per group) of construct 1 (0.7 nmol/kg IP BID) or placebo (20 mM Tris pH 7.5, 135 mM NaCl). Groups were dosed continuously for 28 days. Body weight was monitored continuously throughout the study and fasting blood glucose was measured before and after the treatment period.

### Size Exclusion Chromatography

Size exclusion chromatography (SEC) was performed using a TSK-Gel, G3000 SWXL, 7.8×300 mm HPLC column (Tosoh Bioscience) connected to an HPLC system equipped with an autosampler and UV/VIS detector (Shimadzu). The system was equilibrated in phosphate buffered saline (PBS) at a flow rate of 0.7 mL/min at ambient temperature. For column calibration, a gel filtration standard (BioRad, cat#151-1901) was used. For sample analysis, 20 ul of 1 mg/ml Gcg-XTEN was injected and absorbance was monitored for 20 min using OD214nm.

### Reverse-Phase Chromatography

Reverse phase C18 chromatography (RPC18) was performed using a Phenomenex Gemini 5 µm C18, 100 Å, 4.6×100 mm column (Phenomenex) connected to an HPLC system equipped with an autosampler and UV/VIS detector (Shimadzu). Buffer A was 0.1% TFA in water and Buffer B was 0.1% TFA in 100% acetonitrile. The system was run with a combined flowrate of 1 ml/min. The column was equilibrated in 5% Buffer B at 35°C. The chromatographic separation of Gcg-XTEN was achieved by a linear gradient from 5% to 95% B over 15 minutes. For sample analysis, 10 µl of 1 mg/ml Gcg-XTEN was injected and absorbance was monitored using OD214nm.

### *In vitro* Bioactivity

Samples analysis was performed by Millipore's GPCRProfiler® service using a transfected GcgR cell line (Cat# HTS112C). Calcium flux was monitored in real-time by FLIPR analysis after addition of serial dilutions of Gcg-XTEN or synthetic glucagon.

### Pharmacokinetics

Pharmacokinetic analysis was performed by administration of length constructs to individual cynomolgus monkeys at MPI Research, Inc. (Mattawan, MI). Each compound was dosed subcutaneously at 12 nmol/kg. Plasma concentrations were measured using a sandwich ELISA assay. Briefly, 100 ng of rabbit polyclonal anti-XTEN was immobilized in each well of a polystyrene microtiter plate (Costar 3690, Corning Inc, Corning, N.Y.), followed by blocking with 3% bovine serum albumin (BSA). After 3 washes with PBS, plasma samples were serially titrated across the plate in PBS containing 1% BSA and 0.5% Tween 20. After a 2 hour incubation and washing, the samples were probed by the addition of biotinylated rabbit polyclonal anti-XTEN to each well. After incubation and washing, plates were developed by incubation with horseradish peroxidase-conjugated streptavidin (Thermo Fisher Scientific, Rockford, IL) followed by tetramethylbenzidine substrate (Neogen Corporation, Lexington, KY), then quenched with 0.2 N H_2_SO_4_ and read at 450 nm.

### Pharmacodynamics

Beagle dogs (four per group) were injected subcutaneously with designated doses of synthetic glucagon (American Peptide, Sunnyvale, CA), Gcg-XTEN, or placebo. Animals were fasted for 12 hours preceding dosing and 6 hours following dosing. Blood glucose was tested at designated times before and after dosing using a hand-held glucometer (Walgreens TRUEresult™). Raw data was corrected for systematic bias between human and canine blood by comparison to reference samples across the entire linear range (40–250 mg/dL) measured in parallel at a diagnostic laboratory (Antech Diagnostics).

### Hypoglycemic Challenge

Beagle dogs (four per group, eight total) were subjected to a hypoglycemic challenge model in two phases, separated by a four day washout period. In each phase, the animals were fed three hours prior to injection, then fasted for the remainder of the study. In each phase, groups were injected subcutaneously with 0.6 nmol/kg of Gcg-XTEN or placebo at time zero. In the first phase, hypoglycemic challenge was initiated 6 hours after test article injection by administration of 0.05 U/kg insulin (Novolin-R, Novo Nordisk Pharmaceuticals, Inc.). Hypoglycemic challenge in phase 2 was initiated identically, with the exception of being 12 hours following test article injection. Blood glucose levels were tested at designated times using a hand-held glucometer as above. Blood samples were taken pre-dose and one hour following insulin challenge in all animals. Samples were tested by clinical chemistry to confirm accuracy of the hand-held glucometer readings. In addition, clinical chemistry confirmed significantly elevated insulin levels following hypoglycemic challenge in all groups. Although slightly lower (Table 1) in Gcg-XTEN groups relative to placebo groups, the differences between groups were not statistically significant at either tested timepoint.

### Sequences

#### Construct 1

HSQGTFTSDYSKYLDSRRAQDFVQWLMNTGGEGSGEGSEGEGSEGSGEGEGSEGSGEGEGGSEGSEGEGGSEGSEGEGGSEGSEGEGSGEGSEGEGGSEGSEGEGSGEGSEGEGSEGGSEGEGGSEGSEGEGSGEGSEGEGGEGGSEGEGSEGSGEGEGSGEGSEGEGSEGSGEGEGSGEGSEGEGSEGSGEGEGSEGSGEGEGGSEGSEGEGSEGSGEGEGGEGSGEGEGSGEGSEGEGGGEGSEGEGSGEGGEGEGSEGGSEGEGGSEGGEGEGSEGSGEGEGSEGGSEGEGSEGGSEGEGSEGSGEGEGSEGSGEG

#### Construct 2

HSQGTFTSDYSKYLDSRRAQDFVQWLMNTGGEGGSEGSEGEGSEGSGEGEGGSEGSEGEGSEGSGEGEGGSEGSEGEGGSEGSEGEGGEGSGEGEGSEGSGEGEGSGEGSEGEGGSEGGEGEGSEGGSEGEGSEGGSEGEGGEGSGEGEGGGEGSEGEGSEGSGEGEGSGEGSEG

#### Construct 3

HSQGTFTSDYSKYLDSRRAQDFVQWLMNTGGEGSGEGSEGEGSEGSGEGEGSEGSGEGEGGSEGSEGEGGSEGSEGEGSEGGSEGEGGSEGSEGEGSEGGGEG

#### Construct 4

HSQGTFTSDYSKYLDSRRAQDFVQWLMNTGGEGSGEGSEGEGSEGSGEGEGSEGGSEGEGGSEGSEG

#### Gcg-XTEN

HSQGTFTSDYSKYLDSRRAQDFVQWLMNTGGTSTPESGSASPGTSPSGESSTAPGTSPSGESSTAPGSTSSTAESPGPGSTSESPSGTAPGSTSSTAESPGPGTSPSGESSTAPGTSTPESGSASPGSTSSTAESPGPGTSPSGESSTAPGTSPSGESSTAPGTSPSGESSTAPG
